# Factors contributing to jump heights in two-foot running jumps with and without a basketball

**DOI:** 10.3389/fspor.2025.1597058

**Published:** 2025-06-03

**Authors:** Jun Ming Liu, Antonia Zaferiou

**Affiliations:** Musculoskeletal Control and Dynamics Lab, Department of Biomedical Engineering, Stevens Institute of Technology, Hoboken, NJ, United States

**Keywords:** basketball, jump, ground reaction force, jump height, impulse, dunk

## Abstract

**Introduction:**

Two-foot running jumps (TFRJs) are used by basketball players during games and evaluations for maximum jump height with or without a ball. Prior research on TFRJs performed by volleyball players revealed whole-body kinematics and kinetics variables that contribute to jump height, though it is unknown whether these variables contribute to jump height similarly in TFRJs performed by basketball players, and whether there are differences in how different variables relate to jump height in TFRJs. The objective of this study was to examine the correlation between jump height and whole-body kinematics and kinetics variables in both TFRJs with and without a ball.

**Methods:**

Fifteen male and six female recreational to collegiate basketball players performed TFRJs with and without a basketball with the goal of jumping as high as possible toward an adjustable hoop. Variables of interest include initial forward center of mass (COM) velocity, the angle between a vector from the COM to the heel and horizontal (“plant angle”), COM ascent displacement, upward and backward impulses generated by the first and second legs, and net impulses generated (which also included downward impulse due to body weight).

**Results:**

Jump height had significant positive correlations with initial forward COM velocity, plant angle, COM ascent distance, and net backward and upward impulses in both TFRJs with and without a ball. Jump height also had significant positive correlations with backward and upward impulses generated by the first and second legs in TFRJs without a ball and with second leg upward impulse in TFRJs with a ball.

**Conclusions:**

TFRJs leveraged similar whole-body kinematic and kinetic mechanisms to achieve jump height as other types of running jumps from previous research. Therefore, athletes should aim to develop the physical and technical abilities through resistance training and specific practice support the use of the beneficial biomechanical variables in this study, such as being able to use more initial forward COM velocity, a shallower plant angle, a greater COM ascent distance, and greater overall impulse generation.

## 1 Introduction

**Figure 1:**
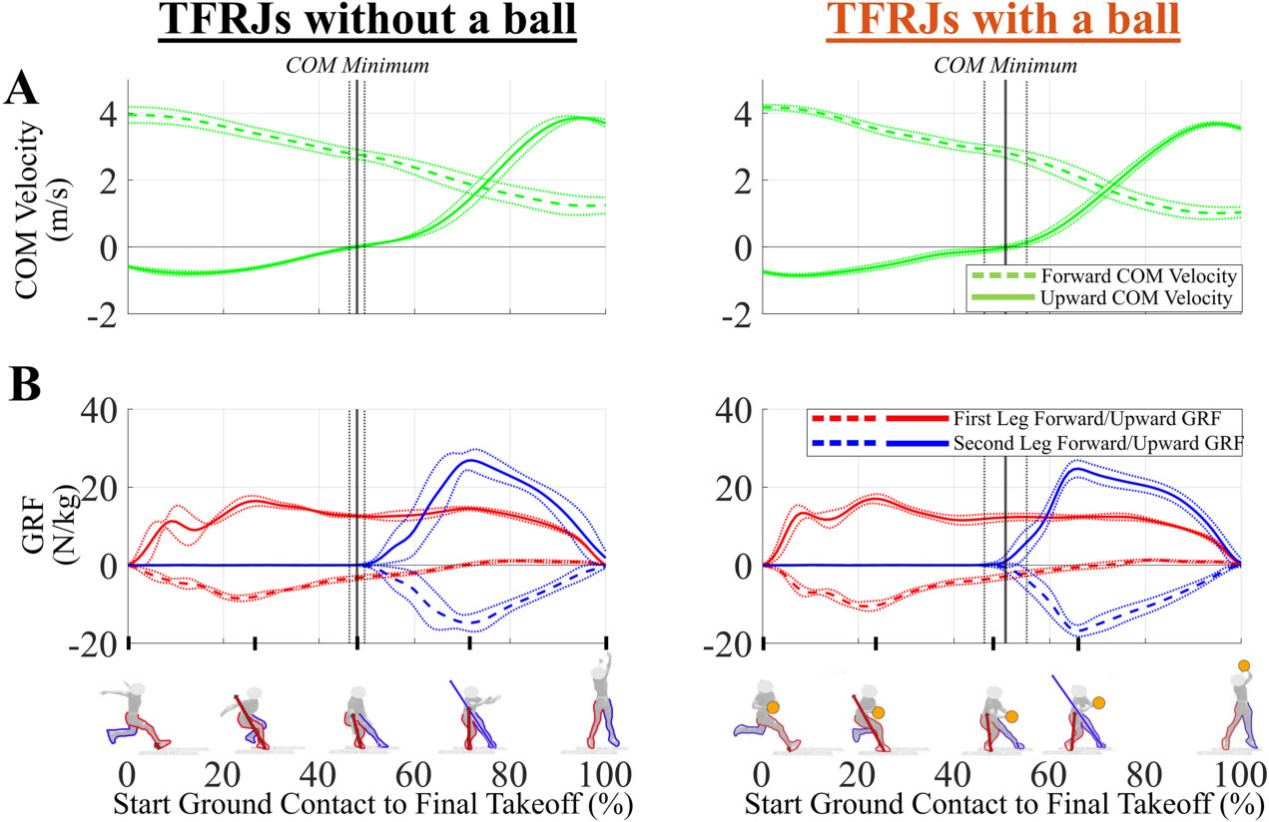
**(A)** Example mean (±1 standard-deviation) center of mass (COM) velocity time-series (green) in the forward (dashed line) and upward (solid line) directions for two-foot running jumps (TFRJs) without a basketball (left) and TFRJs with a basketball (right). **(B)** Example mean (±1 standard-deviation) forward (dashed) and upward (solid) ground reaction force (GRF) time-series for the first leg (red) and the second leg (blue) for TFRJs without a basketball (left) and TFRJs with a basketball (right). Time series began from initial first leg contact (0%) to takeoff (100%). Vertical black solid and dotted lines indicate mean and 1 standard-deviation for timing of COM minimum height, which is the transition from COM descent to COM ascent. Images and data are from participant 19.

“Two-foot running jumps” (TFRJs) involve a running approach followed by sequential ground contacts of each leg and take-off from double support (Figure 1). They may be performed while the athlete controls the basketball and jumps towards the basket to lay up or dunk (“TFRJs with a ball”) or while the athlete tries to catch a ball in the air or defend against a shot attempt (“TFRJs without a ball”). These jumps were used during games (1) and as the preferred method of jumping for maximum height among a cohort of basketball players (2). TFRJs are influenced by both physical and technical factors which contextualize their performance (3), though existing analysis were mostly focused on TFRJs performed by volleyball players. Due to their relevance and specificity, and the lack of existing research on TFRJs performed by basketball players, there is great value in understanding the biomechanical factors that contribute to jump height in TFRJs with and without a basketball. Insights from this study will directly inform physical preparation and technical training that target performance of sports-specific TFRJs, which will be more likely to influence jump performance during ecological contexts compared to “generic” jumps that do not occur often during sport gameplay or practice.

**Figure 2:**
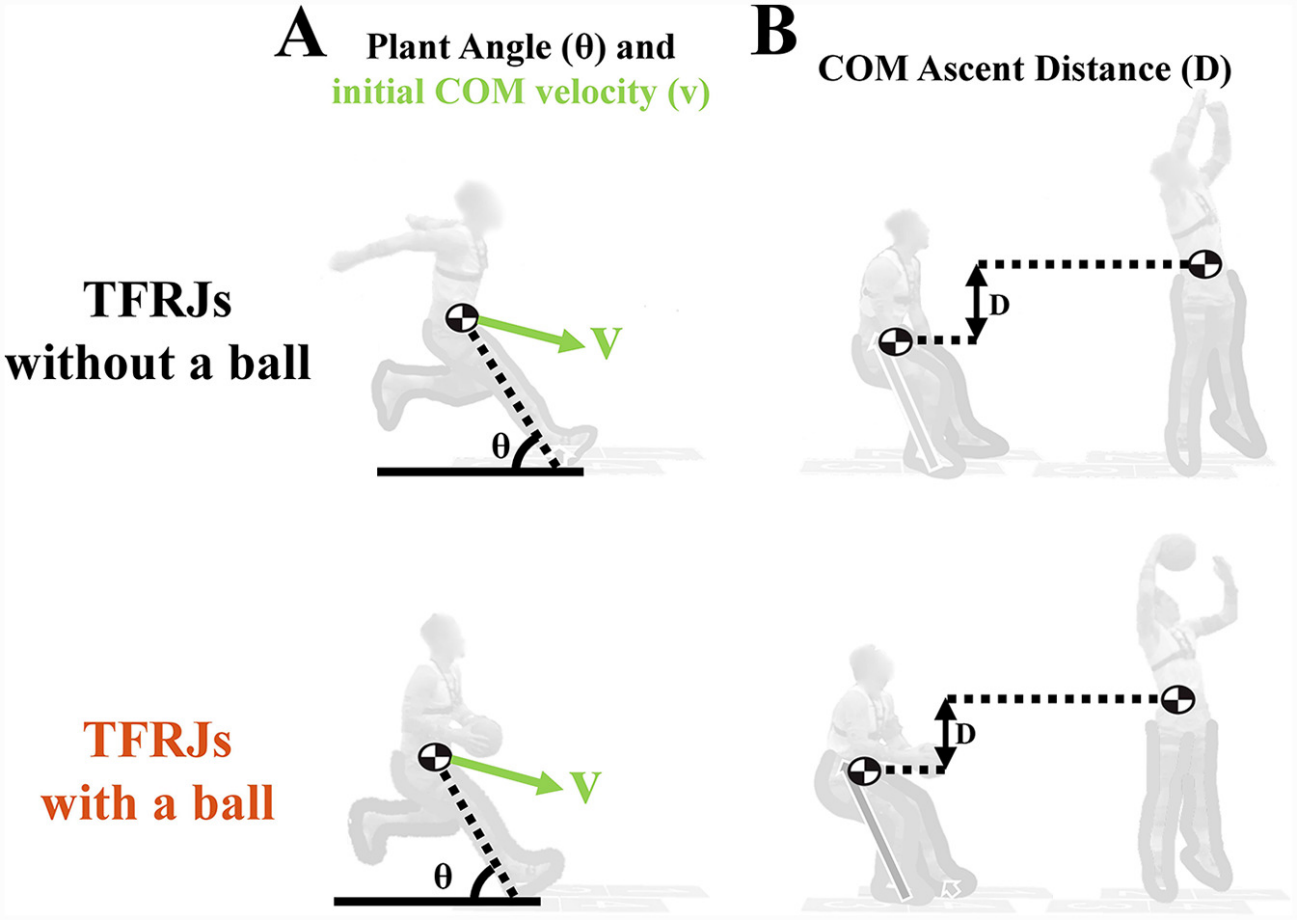
Definitions of whole-body kinematics variables of **(A)** the initial COM velocity (COM velocity at the start of ground contact), plant angle (the angle from the COM to the heel at the start of ground contact), and **(B)** the COM ascent distance (vertical distance from the COM minimum point until the COM height at takeoff). Images and data are from participant 1 performing TFRJs without a ball (top) and TFRJs with a ball (bottom).

Existing research on biomechanical variables that contribute to jump height in TFRJs was limited to TFRJs performed by volleyball players. Jump height, measured by COM upward velocity at takeoff, is causally and directly controlled by the initial downward COM velocity and the net upward impulse through the impulse-momentum relationship. Other kinematics and kinetics variables were investigated in prior literature, though they mainly contribute to jump performance through indirect means by augmenting net upward impulse generation. Of the whole-body kinematics variables, initial center of mass (COM) horizontal velocity, anterior limb positioning relative to the body's COM, and vertical COM displacement during the ascent (referred to as the “COM ascent distance”) were most frequently investigated (Figure 2). In TFRJs, initial horizontal COM velocity from the running approach correlated with jump height in most prior studies (3–5) apart from one (6). COM velocity before ground contact allows athletes to leverage the stretch-shortening cycle in the lower limb musculature better (7, 8). More anterior positioning of the first leg in front of the body's COM correlated with jump height in TFRJs in volleyball (3, 5). This may be due to the anterior positioning of the foot relative to the body's COM benefiting impulse generation (3, 9, 10). Greater COM ascent distance correlated with jump height, because it affords a longer duration of upward COM acceleration (3, 5, 11).

Only one study examined and found significant correlations between jump height and total upward impulse of both legs and the upward and backward impulses of the second leg (10). A significant positive correlation between jump height and net vertical impulse is expected in the present study. Other relationships may emerge between jump height and other impulse variables due to the different patterns of impulse generation during TFRJs performed by basketball players (9) compared to TFRJs performed by volleyball players (3, 10).

TFRJs may be performed with or without a basketball in practice and gameplay scenarios. So far, TFRJs have rarely been investigated in the context of basketball players, and the influence of ball control on the contribution of initial forward COM velocities and impulse generation toward jump height is unknown. The demand of dribbling the ball before the takeoff phase likely reduces the initial COM velocities, as basketball players were found to run slower with vs. without a ball (12). This may reduce the contribution of the initial forward COM velocity toward jump height in TFRJs with a ball. Net upward impulse generated during TFRJs with a ball should still be directly and mechanically relevant to jump height, though the need to control the ball during the total ground contact phase may alter arm swing, which has been linked to differences in upward impulse generation in stationary countermovement jumps with and without arm swing (13–15). Still, it remains unknown whether different relationships between jump height and whole-body kinematics and kinetics variables would be observed between TFRJs with and without a basketball.

The purpose of this study was to investigate the correlations between jump height and whole-body kinematics and kinetics variables in both TFRJs with and without a ball and explore differences between conditions. It was hypothesized that jump height would have significant positive correlations with initial forward COM velocity, COM ascent distance, anterior positioning of the first leg relative to the body's COM, net upward and backward impulses generated by both legs, and upward and backward impulses generated by each leg.

## 2 Materials and methods

### 2.1 Participant information

21 basketball players (15 males and 6 females, mean (standard deviation) age of 22.5 (4.40) years, 1.81 (0.1) m height, 80.21 (10.15) kg weight) with recreational or college-level basketball experience were recruited for this study. Participants were informed of the study procedure and provided their informed consent to volunteer for this study that was approved by the Institutional Review Board on human subject research at Stevens Institute of Technology. Participants were included only if they met all of the individual criteria: (1) they do not have any current musculoskeletal injury, (2) they competed at the college-level in basketball or play basketball recreationally for at least 3 h a week or specifically practice TFRJs weekly, (3) self-reported comfortability performing TFRJs with and without a ball, and (4) self-reported a jump height of 0.5 m for male participants and 0.4 m for female participants. The performance criteria were selected based on the stationary countermovement jump heights in prior literature (15, 16), and TFRJs were expected to result in higher jump heights compared to stationary countermovement jumps (2, 5). Sample size was computed in G*power 3.1.9.7 using the Correlation: Point Biserial model in the *t*-test family. The tests were one-tailed with the effect size of 0.5 based on the correlation coefficients from previous research on jump height and impulse variables (10), a desired power of 0.80, and *α* = 0.05. This resulted in a required sample size of 21.

### 2.2 Data collection procedures

**Figure 3:**
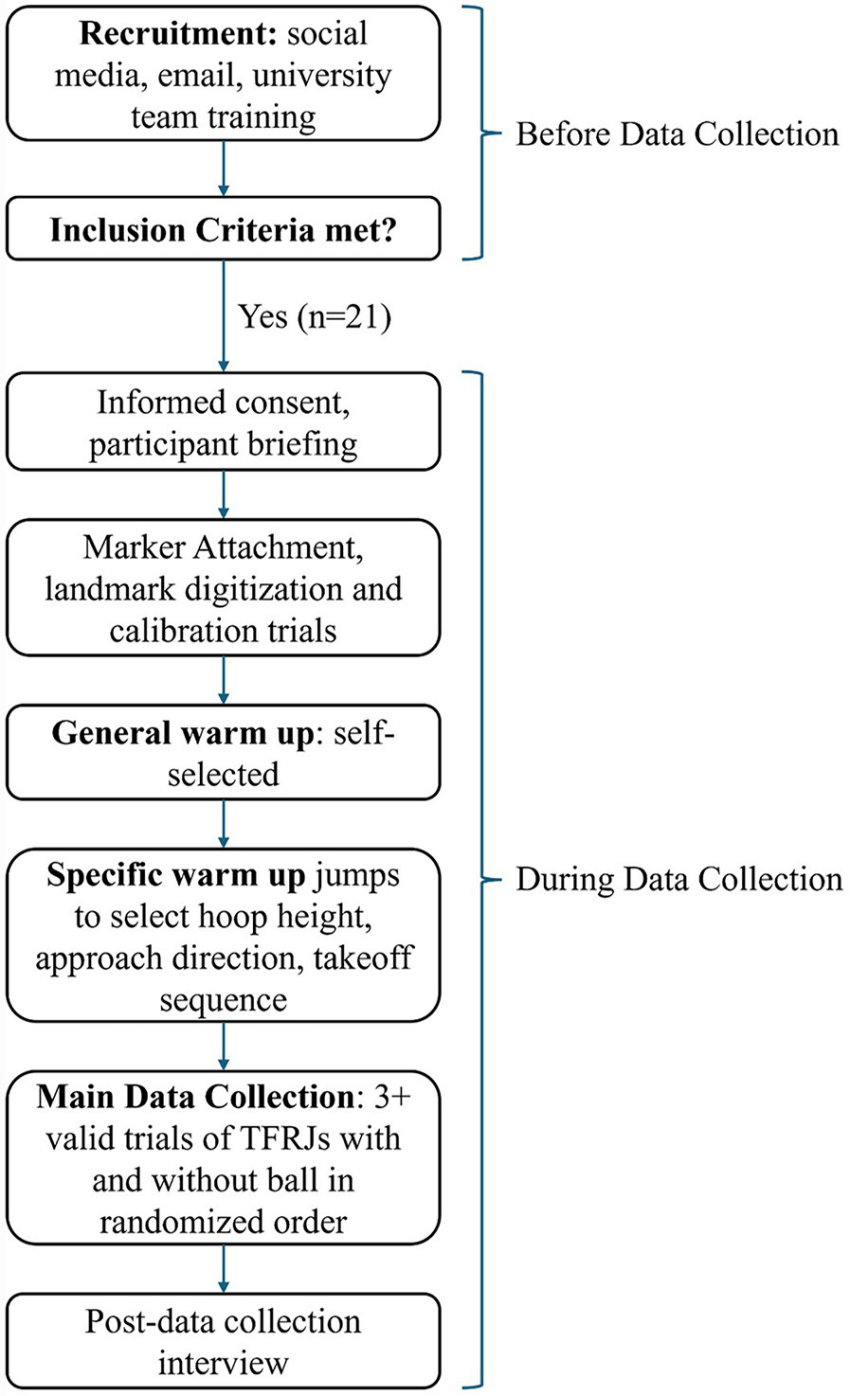
Flow chart of study design from participant recruitment to the data collection.

A study design flow chart is included in Figure 3. Participants were instructed to wear their preferred shoes to prioritize comfort. To capture 3D kinematics, rigid clusters with four infrared reflective markers were attached to the torso, bilateral upper arm, forearm, thigh, shank, and foot segments. A headband with 4 individual markers was worn to the head. Individual markers were attached to the bilateral anterior and posterior superior iliac spine on the pelvis and the second and fifth metacarpal of the hand, as well as the back of the hand. Anatomic landmarks were then digitized using a digitizing probe following previously described procedures (9). Participants performed self-selected warmups after marker attachment and landmark digitization. They then performed practice trials of TFRJs with and without a ball toward a height-adjustable basketball hoop from 4.57 m away, consistent with the NBA combine test distance. During the practice trials, the height of the hoop was selected per participant (2.40–3.05 m) to elicit the intention to jump as high as possible. Participants were free to select their direction of approach and their takeoff sequence to be used for both TFRJs with and without a ball to ensure they were using a preferred and well-practiced approach, as they are skilled athletes performing two goal-directed tasks in an observational study. Participants could either use a Right-Left sequence or a Left–Right sequence though had to be consistent between the conditions. No other standardization was enforced. Participants could either use a Right-Left sequence or a Left-Right sequence. Further information about each participant is included in the Supplementary Document. For TFRJs without a ball, participants were instructed to jump as high as they could and tap their preferred limb on the hoop as high as they could. For TFRJs with a ball, participants were instructed to dribble the ball during the running approach, jump as high as they could while holding the ball, and dunk the basketball into the hoop with their preferred limb if they were able. Regardless of whether the dunk was successful, the trial was included in the biomechanical analysis of the takeoff. An interview was conducted after the data collection to understand each participant's jump training history (Supplementary Document). The International Basketball Federation 3 × 3 official basketball was used (17). Participants were provided with self-selected rest time between trials in addition to transition times between trials of at least 1 min. Participants performed at least three trials that had valid force plate contact by each leg and were rated as representative by the participants (9). A total of 374 trials were recorded, 119 were excluded due to improper force plate contacts and 15 were excluded due to low participant ratings.

3D kinematics were captured at 250 Hz using 22 cameras from Optitrack Motive (NaturalPoint Inc., Corvallis, Oregon, USA). 3D kinetics were captured at 1,000 Hz using four in-ground Bertec force plates (Bertec, Columbus, Ohio, USA). Motive 3.0 motion capture software (NaturalPoint Inc., Corvallis, Oregon, USA) was used to time-synchronize the kinematics data with the kinetics data from the force plates.

### 2.3 Data processing

The data were imported into MATLAB R2023b (RRID: SCR_001622, MathWorks, Natick, Massachusetts) for processing. Raw GRF and marker data were filtered using a 4th-order Butterworth filter with a cutoff frequency of 35 Hz to remove noise from possible force plate vibrations while keeping as much of the original signal as possible (18, 19). These filtered data were inspected visually to ensure that the filters were appropriate.

The global orthogonal axes were defined with respect to the trajectory of the body's COM during the flight phase for each trial of each participant (9). The forward axis was the average horizontal forward COM trajectory direction during flight, the upward axis was the lab's global upward axis, and the leftward axis was the cross product of the upward and forward axes. The phases of interest included the total ground contact phase that started from the first leg's ground contact until the final takeoff and was normalized to 101 data points from 0% to 100% (Figure 1). The takeoff and ground contact of each leg were determined using an upward GRF threshold of 10 N. The total ground contact phase was further divided into the COM descent subphase from the time indices of the first leg's initial ground contact until the COM's lowest vertical position (“COM minimum point”) and the COM ascent subphase from COM minimum point until the final takeoff.

The digitized anatomic landmarks defined the end points of each body segment according to de Leva and allowed for the computation of whole-body COM position (20). Joint center locations were computed per recommendations from the literature: shoulder joint centers (21); hip joint centers (22); elbow, wrist, knee, and ankle joint centers (23). Impulses generated by each leg were computed as the time integral of the GRFs through the phases of interest and normalized by body mass (24). Net upward impulse was computed as the sum of each leg's upward GRF impulses minus the downward impulse due to body weight (the product of the duration of the phase of interest and body weight). Initial forward and downward COM velocities were calculated at initial ground contact of the first leg, and jump height was calculated from upward COM velocity at takeoff (25). COM ascent distance was computed as the difference in vertical positions between the COM height at takeoff and COM's minimum position (Figure 2). Plant angle was computed using the vector from the COM to the heel marker and the horizontal backward vector at initial ground contact of the first leg (Figure 2).

### 2.4 Statistical analyses

Statistical analysis was performed in MATLAB R2023b (MathWorks, Natick, Massachusetts). The representative value of the biomechanical variables for each participant in each condition is the mean of the valid trials in each condition. The normality of the data was examined using the Shapiro–Wilk test. The relationships between jump height and the biomechanical variables of interest from either TFRJs with a basketball or TFRJs without a basketball were examined using Pearson's product-moment if the data were normally distributed or Spearman's rank correlation coefficients if the data were not normally distributed (*α* = 0.05). Holm's adjustment for multiple comparisons was performed in MATLAB (26).

## 3 Results

**Figure 4:**
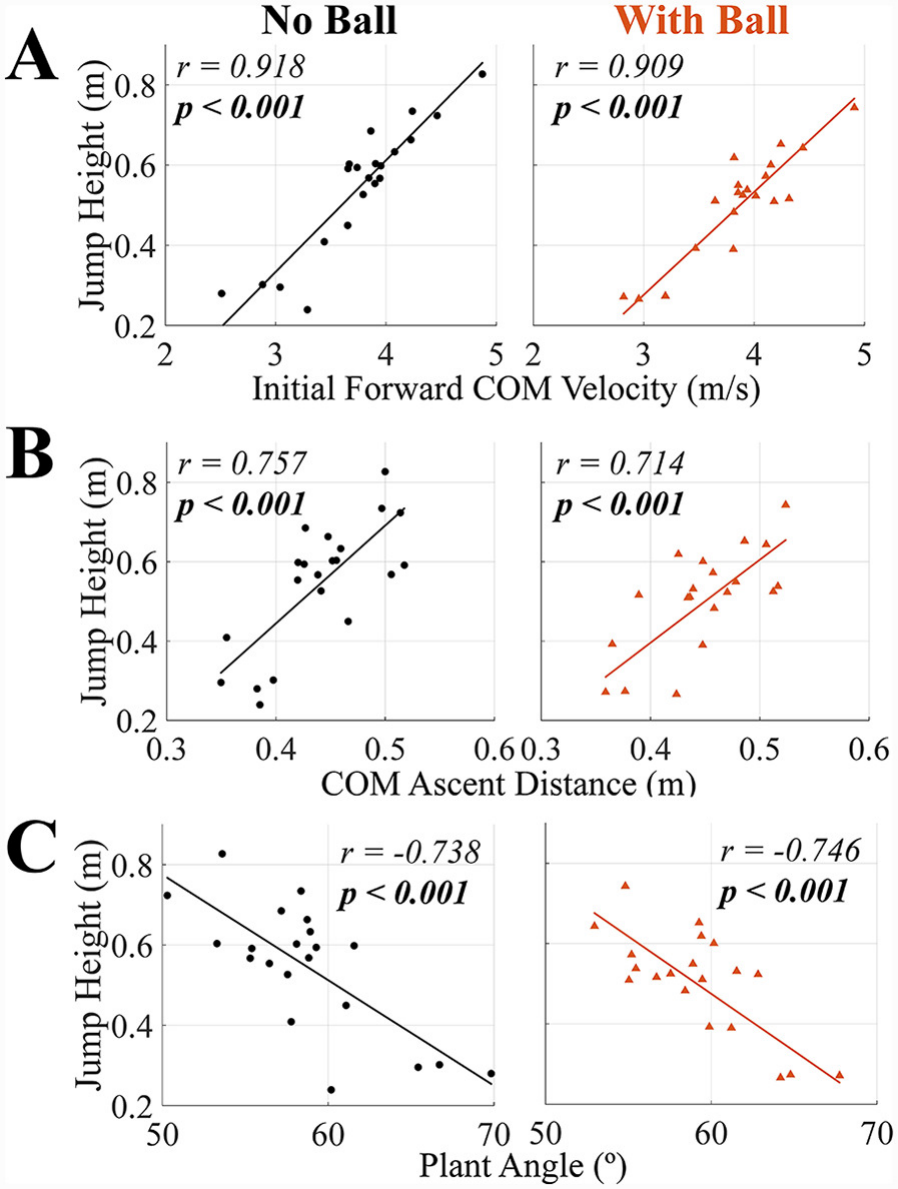
Correlation coefficient and *p-*values for the Pearson's correlations between jump height and **(A)** initial forward COM velocity, **(B)** COM ascent distance, and **(C)** plant angle in TFRJs with and without a basketball. *p-*values bolded if significant (*α* = *0.05*). All variables presented here are normal and only Pearson's correlation was used.

**Figure 5:**
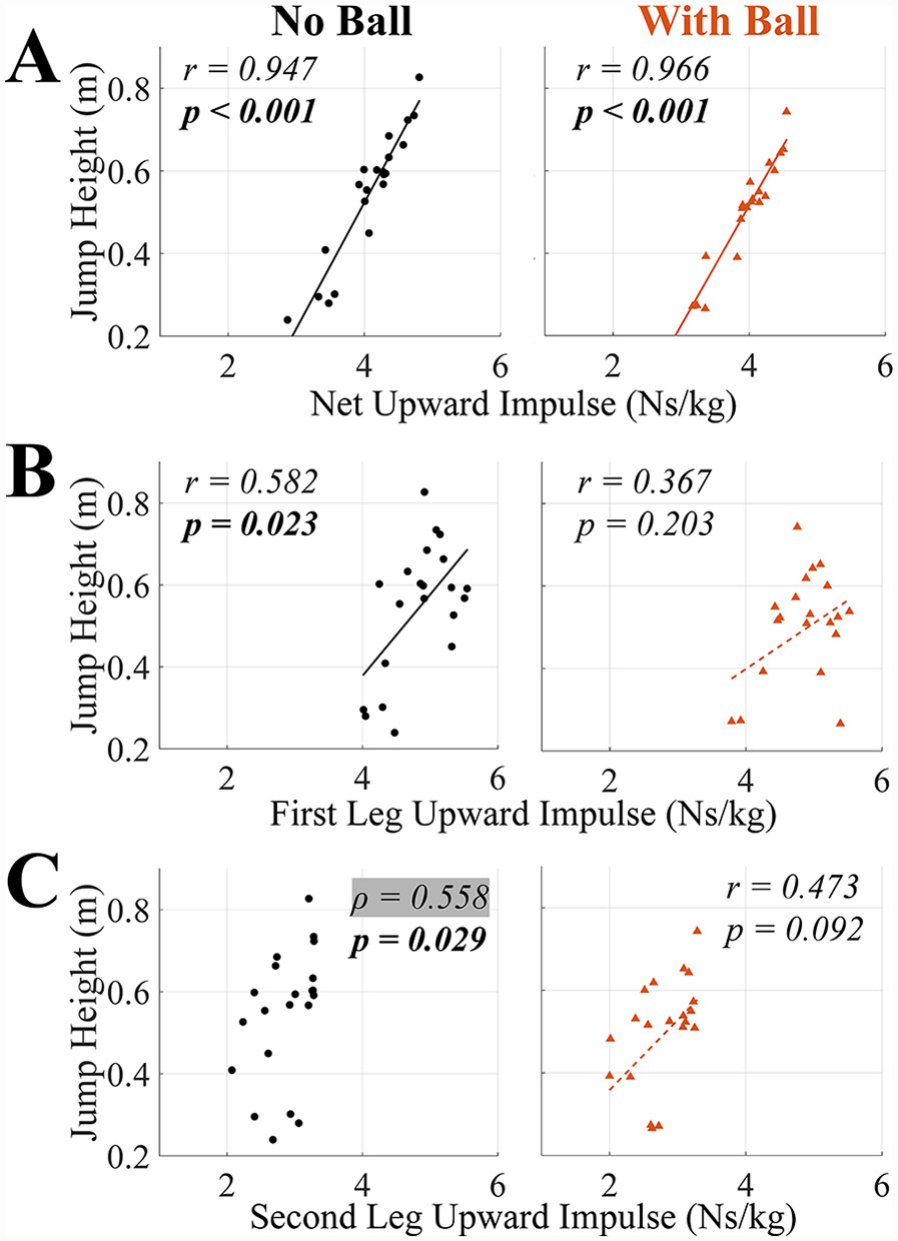
Correlation coefficient (r if the data is normally distributed and Pearson's correlation was used and *ρ* if the data is not normally distributed and Spearman's rank correlation was used) and *p-*values for the correlations between jump height and **(A)** net upward impulse, **(B)** first leg upward impulse, and **(C)** second leg upward impulse in TFRJs with and without a basketball. *p-*values bolded if significant (*α* = *0.05*). Line of best fit is solid if Pearson's correlation was significant and dashed if not. The use of Spearman's rank correlation is indicated by highlighting *ρ* in grey and do not have a line of best fit visualized.

**Figure 6:**
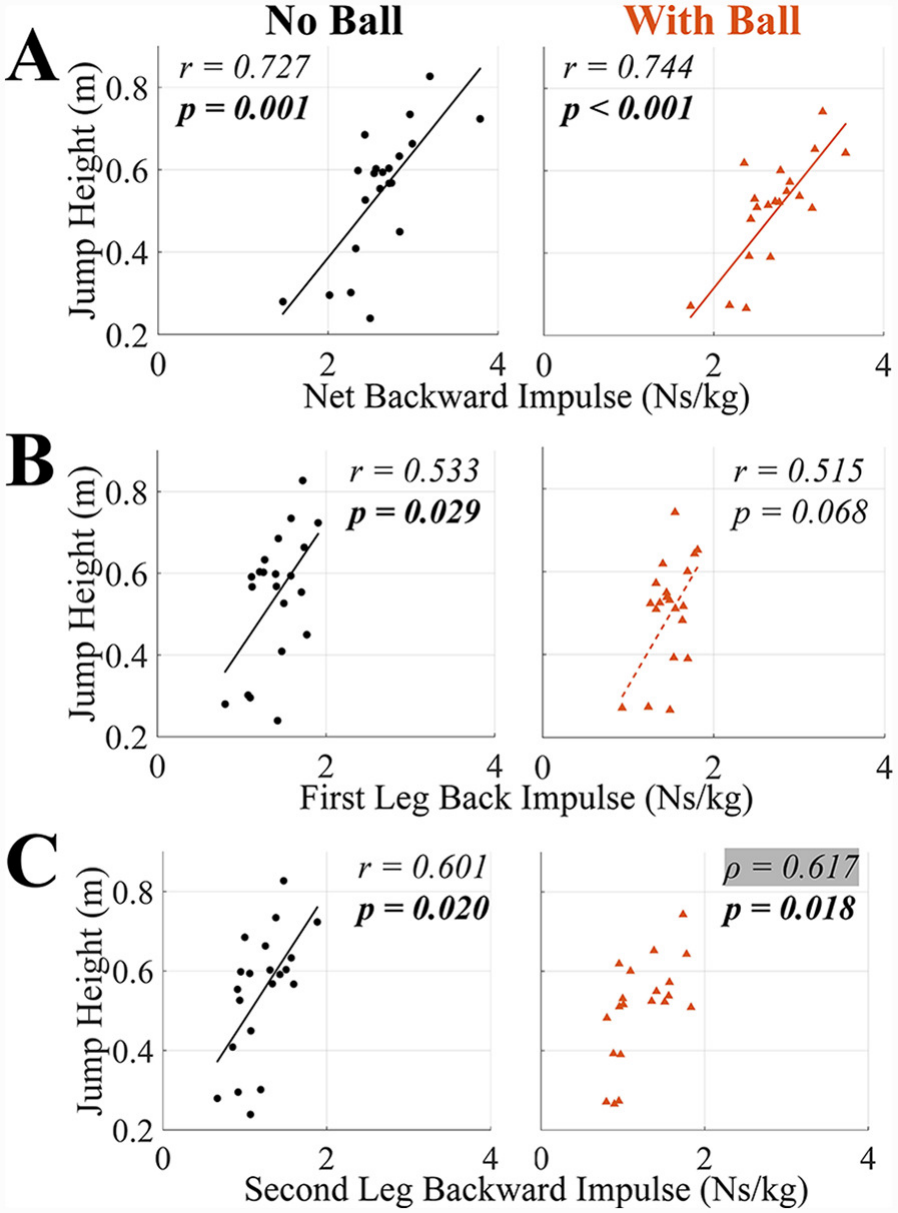
Correlation coefficient (r if the data is normally distributed and Pearson's correlation was used and *ρ* if the data is not normally distributed and Spearman's rank correlation was used) and *p-*values for the correlations between jump height and **(A)** net backward impulse, **(B)** first leg backward impulse, and **(C)** second leg backward impulse in TFRJs with and without a basketball. *p-*values bolded if significant (*α* = *0.05*). Line of best fit is solid if Pearson's correlation was significant and dashed if not. The use of Spearman's rank correlation is indicated by highlighting *ρ* in grey and do not have a line of best fit visualized.

For the hypothesized variables with significant results (*p* *<* *0.05*), the correlation coefficients ranged from 0.533 to 0.966. For whole-body kinematics variables, jump height had significant positive correlations with initial forward COM velocity and COM ascent distance in both TFRJs without and with a basketball (*p* *<* *0.002* for all), and significant negative correlations with plant angle (more anterior positioning) in both TFRJs with and without a basketball (*p* *<* *0.001* for both) (Table 1, Figure 4). For upward impulse variables, jump height had significant positive correlations with net upward impulse in both TFRJs without and with a basketball (*p* *<* *0.001* for both) and first and second leg upward impulse in TFRJs without a basketball (*p* *=* *0.023* and *p* *=* *0.029*, respectively) (Table 1, Figure 5). For backward impulse variables, jump height had significant positive correlations with net backward impulse in both TFRJs without and with a basketball (*p* *<* *0.001* for both), first and second leg backward impulse in TFRJs without a basketball (*p* *=* *0.029* and *p* *=* *0.020*, respectively), and second leg backward impulse in TFRJs with a basketball (*p* *=* *0.018*) (Table 1, Figure 6).

**Table 1:** Group-level mean (±1 standard-deviation), correlation coefficient, and *p*-value of the whole-body kinematics and impulse variables against jump height in TFRJs with and without a basketball. *p*-value bolded if significant (*α* = 0.05). Net upward impulse also accounted for the downward impulse due to body weight, such that it does not equal to the sum of upward impulse generated by both legs. Variables with non-normal distributions that required the use of Spearman's rank correlation coefficient are highlighted in gray.

Variable name	TFRJs without a basketball			TFRJs with a basketball		
	Mean (S.D.)	Correlation coefficient	*p*-value	Mean (S.D.)	Correlation coefficient	*p*-value
Jump height (m)	0.62 (0.16)	—	**—**	0.58 (0.15)	—	—
Initial forward COM velocity (m/s)	3.76 (0.53)	0.918	**<0.001**	3.83 (0.51)	0.909	**<0.001**
COM ascent distance (m)	0.44 (0.05)	0.757	**<0.001**	0.45 (0.05)	0.714	**0.002**
Plant angle (°)	58.76 (4.52)	−0.738	**<0.001**	59.46 (3.76)	−0.746	**<0.001**
Net upward impulse (Ns/kg)	4.07 (0.50)	0.947	**<0.001**	3.91 (0.48)	0.966	**<0.001**
First leg upward impulse (Ns/kg)	4.84 (0.47)	0.582	**0.023**	4.83 (0.47)	0.367	0.203
Second leg upward impulse (Ns/kg)	2.88 (0.39)	**0.558**	**0.029**	2.78 (0.40)	0.473	0.092
Net backward impulse (Ns/kg)	2.62 (0.46)	0.727	**0.001**	2.69 (0.42)	0.744	**<0.001**
First leg backward impulse (Ns/kg)	1.41 (0.28)	0.533	**0.029**	1.47 (0.21)	0.515	0.068
Second leg backward impulse (Ns/kg)	1.21 (0.30)	0.601	**0.020**	1.21 (0.34)	**0.617**	**0.018**

Additional exploratory analyses were performed between jump height and other variables such as backward and upward impulses generated through the subphases, total ground contact and subphase durations, and average backward and upward GRFs (Supplementary Documents). Notable findings include that jump height had significant positive correlations with net upward and backward impulse generated during the COM ascent subphase (*p* *<* *0.002* for all) and each leg's average upward and backward GRFs (*p* *<* *0.037* for all) for both TFRJs with and without a ball, and no significant correlations with any of the total ground contact phase or subphase durations.

## 4 Discussion

This study examined the relationship between jump height and whole-body kinematic and kinetic variables in both TFRJs with and without a basketball. Jump height had significant positive correlations with initial forward COM velocity, COM ascent distance, plant angle, and net upward and backward impulses in both TFRJs with and without a basketball. Jump height had significant positive correlations with impulses generated by each leg in TFRJs without a basketball, but only second leg upward impulse in TFRJs with a ball.

The correlations between jump height and initial forward COM velocity and plant angle in both TFRJs with and without a basketball were consistent with prior research on TFRJs in volleyball (3–5), and contrasted with one prior study (6). At the group-level, athletes with greater jump heights used faster initial forward COM velocities and shallower plant angles (more anterior positioning of the first leg relative to the body's COM). These strategies may be effective for the lower limb musculature to generate more backward and upward impulses. However, the contribution of initial forward COM velocity towards vertical jump height requires the athlete to effectively generate impulses (3, 10), for which a smaller plant angle (more anterior positioning of the first leg) could be beneficial (3, 27). For each athlete, there may be a “maximum controllable velocity” that will result in optimized jump height at any time point, limited by the athlete's strength and structure (28). In terms of practical implications, athletes should perform specific practice of TFRJs to develop the coordination to position the limbs around the body's COM to generate the required impulses. Athletes may also experiment with initial forward COM velocities that may be greater than their “maximum controllable velocity” while using smaller plant angles as a means of developing better deceleration capabilities and/or perform other horizontal deceleration training (29). Progressive resistance training to enhance lower limb joint strength capacity (i.e., eccentric training of the hip and knee extensors and ankle plantarflexors) may also be applied to support the generation of impulses against the ground to redirect the body's momentum (30).

Jump height's correlation with net backward impulses helps contextualize how athletes leverage the initial forward COM velocity to enhance their jump height. The significant positive correlation between jump height and net backward impulses in both TFRJs with and without a ball was not mirrored by the prior research (10). However, this may relate to the greater overall net backward impulse generated and also greater jump heights by the athletes in this study compared to the prior study (10), which may relate to how the athletes in this study were able to leverage the initial forward COM velocity for greater overall impulse generation and jump performance. However, the previous study did not include the initial conditions from the running approach. The generation of backward impulses plays a support role for jump performance and will likely increase as athletes become accustomed to faster initial forward COM velocity, assuming they can achieve similar takeoff angles. However, during the learning process of TFRJs where the initial forward COM velocity may exceed the athlete's ability to generate sufficient backward impulses to control the body's velocity, shortening the running approach and reducing the initial forward COM velocity may be beneficial for performance in the short-term, until the athlete's deceleration capabilities improve (28).

The correlation between jump height and COM ascent distance was consistent with prior research on TFRJs in volleyball (3, 5), high jump (11, 31), and stationary countermovement jumps (32, 33). The COM vertical height at takeoff is unlikely to be modified, thus, COM ascent distance may be increased by having a lower COM minimum vertical position. The lower COM minimum vertical position may be achieved through a combination of a lower initial COM height, or greater initial downward COM velocity. During stationary countermovement jumps, a lower COM minimum position can only be achieved through more initial downward COM velocity given that the jumping motion begin from stationary standing (32, 33). In contrast, running jumps afford lowering the body's COM height during the running approach without excessive increases in initial downward COM velocity (11). In the high jump, it was believed that faster initial downward COM velocity may be mechanically disadvantageous because a sub-phase of the net upward impulse generated could be dedicated to reducing downward COM velocity rather than increasing upward COM velocity (11, 34). However, initial downward COM velocity is unavoidable due to the flight phase before the start of initial ground contact and may provide a helpful strategy for some to rapidly load their lower limb musculature to generate greater upward impulse (8, 35), should their lower limb musculatures be able to leverage the downward COM velocity. To increase COM ascent distance, athletes can strategically alter COM height during the running approach and/or modify their initial downward COM velocity. As previously suggested, progressive resistance training could also be used to ensure that lower limb musculature can maintain joint torque and upward GRF generation in potentially more demanding positions (36).

The significant positive correlations between jump height and upward impulses in TFRJs were expected yet warrant further discussion. The initial downward COM velocity and net upward impulse both directly contribute to upward COM velocity at takeoff, and thus jump height, through the impulse-momentum relationship. However, only net upward impulse had a significant positive correlation with jump height, meaning that it was the major contributor towards jump performance at the group level. This was consistent with prior research and unsurprising. Even though the upward impulses generated by each leg also directly contribute to net upward impulse, their correlations with jump height captured *how* strategies of impulse generation relate to performance. In TFRJs without a ball, the correlation between jump height and second leg upward impulse was consistent with the prior study (10). This corresponded with the second leg's ground contact overlapping with the COM ascent subphase, resulting in nearly all second leg upward impulse contributing to an increase in the upward COM velocity and thus jump height. In TFRJs without a ball, jump height also correlated with the first leg upward impulse. This was due to the much larger contribution of the first leg towards upward impulse generation and thus upward acceleration of the body's COM in this cohort of basketball players (9), unlike the prior study which had found that the second leg was the major contributor of upward impulse generation (10). In practice, upward impulse generation may be enhanced through a combination of specific practice of TFRJs, general plyometric exercises that challenge and develop upward GRF generation abilities, and progressive resistance training that targets lower limb joint strength capabilities. Upward impulse may also be indirectly modified through differences in initial conditions such as greater initial forward and/or downward COM velocities, or greater COM ascent distance through a lower running approach and/or initial downward COM velocity.

Interestingly, the impulse generation patterns reported in two prior studies were different from each other and different from the present study (3, 10). This highlighted that TFRJs, by their nature of having subsequent ground contacts of each leg, allowed for coordination of each leg to distribute the responsibilities of impulse generation in multiple ways. Even though it is too early to declare any specific patterns of impulse generation as being superior for jump height, the importance of overall backward and upward impulse generation towards jump performance was demonstrated by the strong correlation between jump height and net backward and upward impulse in both TFRJs with and without a ball. The observed impulse generation patterns may be affected by each athlete's muscular strength, structure, training history, and coordination (37), and may all be valid so long as enough total impulse was generated to redirect the body's momentum upward.

TFRJs performed by basketball players provide a unique context to investigate the influence of additional task constraints of ball control on performance. These results complement our ongoing research about COM velocity and impulse differences between TFRJs with and without a basketball. In the present study, the lack of significant positive correlation between jump height and upward impulses generated by either the first or second leg in TFRJs with a ball relates to the observations in the complementary research directly comparing TFRJs with vs. without a ball (38). Specifically, there were participant-specific impulse generation patterns between TFRJs with and without a ball. Thus, with this additional context, the present study's findings suggest that specific patterns of backward or upward impulse generation by each leg may be of secondary importance for jump performance. Instead, the predominant importance of generating sufficient *net* upward and backward impulses is evident by the much stronger correlations between jump height and net upward and backward impulses. The biomechanical differences between TFRJs with vs. without a ball warrant further direct research.

Though not a main research question of this study, we preliminarily explored correlations between jump height and the biomechanical variables when stratified by biological sex (Supplementary Document). This is important due to historical underrepresentation of female athletes in sports biomechanics research (39–41). In this small cohort, male athletes jumped higher compared to female athletes in both TFRJs with and without a basketball, with jump heights of the male and female athletes being similar to those reported in prior studies (3, 10). These initial exploratory findings are severely limited by sample size (*n* = 6 for females vs. the power analysis requiring 21 participants) and a smaller range of observed jump heights. The present study's interview data preliminarily suggests that the different results in the female athletes' data may also be due to differences in the amount of specific practice that the athletes have performed (40). The highest jumping female reported that she specifically and *regularly* practices TFRJs to maximize jump height and dunk a basketball (Supplementary Document). Similarly, the highest jumping male athletes reported that they specifically practiced TFRJs for maximum jump height regularly compared to lower-jumping male athletes. Given the greater jump heights in TFRJs compared to stationary countermovement jumps and the numerus biomechanical differences between TFRJs when compared to stationary countermovement jumps (2, 5), specific practice of TFRJs for maximum height both with and without a basketball should be prescribed for both male and female athletes, both as a way to open up more scoring and defensive options during games, but also as a method of athletic development.

This study has several limitations that guide future research. Firstly, the results of the correlation analysis are limited by the ranges of jump performance displayed by the athletes, and more elite athletes may display different movement patterns. Secondly, due to a constraint of lab space, the participants were limited to a shorter approach distance of 4.57 m (15 feet), which is the distance from the free throw line to the basket and equal to the NBA combine distance used for jump performance evaluations. More advanced athletes may jump higher with a longer approach distance, though future studies should further investigate the impact of running approach distance on jump performance during TFRJs. Additionally, there may be spatiotemporal or contextual constraints during basketball gameplay that enforce a shorter approach, and the athletes will have to maximize jump height despite the shorter approach distance. Thirdly, only a limited number of female participants were included despite consistent and specific efforts during the recruitment process over three years. We encourage future research to be specifically designed to evaluate female-only TFRJs or possible sex differences. Lastly, only TFRJs were examined in this study. While there is value in studying TFRJs as they allow for concurrent coordination of each leg to redirect the body's momentum and that they are underrepresented in running jump research despite their use in games and evaluations, one-foot running jumps are also important to understand in basketball biomechanics research. One-foot running jumps are still viable for layups and dunks during basketball, and future research should compare different jump types during different gameplay and testing contexts to discover whether the double support phase of TFRJs may be more adaptable for jumps with varying demands of momentum regulation.

In conclusion, this study revealed the contribution of initial forward COM velocity, COM ascent distance, plant angle, and the net backward and upward impulses toward jump height in TFRJs with and without a basketball. The whole-body kinematics and kinetics variables strongly relate to jump height in basketball players in both TFRJs with and without a ball, though there were differences between TFRJs with and without a ball in the correlations between jump height and sub-system level variables such as impulse generation by each leg.

## Data Availability

The datasets presented in this article are not readily available because the raw data supporting the conclusions of this article will be made available by the authors, upon request, for research participants who opted-in to publicly share their deidentified data. Requests to access the datasets should be directed to antonia.zaferiou@stevens.edu.
