# Supplementary material for: Factors contributing to jump heights in two-foot running jumps with and without a basketball
Source: Front Sports Act Living. 2025 Jun 3;7:1597058. doi: 10.3389/fspor.2025.1597058 (PMC12180369; doi:10.3389/fspor.2025.1597058)
Supplement: Supplementary file 1 [file Table1.docx]

Supplementary Material

# Supplementary Tables Links

[Table 1: Participant Information](#table1)

[Table 2: Participant Number key with prior study](#table2)

[Table 3: Other Variables vs. Jump Height](#table3)

[Table 4: Results stratified by sex](#table4)

[Table 5: Participant Post-data Collection Interview Responses](#table5)

**Supplemental Document Table 1:** Participant and group biological sex, height, weight, takeoff sequence.

| **Subject** | **Sex** | **Height** | **Weight** | **Takeoff Sequence** | **Mean (S.D.) Jump Height (m)** | |
| --- | --- | --- | --- | --- | --- | --- |
|  |  |  |  |  | **TFRJs without a basketball** | **TFRJs with a basketball** |
| 1 | m | 1.89 | 94.04 | RL | 0.77 (0.03) | 0.72 (0.03) |
| 2 | m | 1.86 | 81.38 | LR | 0.73 (0.01) | 0.69 (0.02) |
| 3 | m | 1.82 | 81.81 | LR | 0.65 (0.01) | 0.57 (0.02) |
| 4 | m | 1.86 | 81.84 | RL | 0.6 (0.03) | 0.55 (0.02) |
| 5 | f | 1.63 | 63.61 | LR | 0.51 (0.02) | 0.5 (0.04) |
| 6 | f | 1.84 | 70.93 | RL | 0.39 (0.01) | 0.35 (0.01) |
| 7 | m | 1.61 | 66.91 | RL | 0.62 (0.01) | 0.63 (0.02) |
| 8 | m | 1.82 | 90.99 | RL | 0.75 (0.03) | 0.67 (0.01) |
| 9 | m | 1.87 | 84.02 | LR | 0.63 (0.02) | 0.6 (0.03) |
| 10 | m | 1.86 | 95.61 | RL | 0.54 (0.02) | 0.52 (0.02) |
| 11 | m | 1.95 | 93.39 | RL | 0.6 (0.02) | 0.59 (0.02) |
| 12 | m | 1.83 | 92.5 | LR | 0.68 (0.08) | 0.56 (0.09) |
| 13 | m | 1.7 | 68.34 | LR | 0.85 (0.01) | 0.8 (0.01) |
| 14 | f | 1.75 | 72.32 | LR | 0.36 (0.03) | 0.35 (0.01) |
| 15 | m | 1.89 | 75.62 | LR | 0.67 (0.02) | 0.61 (0.01) |
| 16 | m | 1.93 | 92.54 | LR | 0.84 (0.02) | 0.78 (0.02) |
| 17 | m | 1.89 | 84.91 | RL | 0.78 (0.01) | 0.73 (0.01) |
| 18 | m | 1.89 | 78.54 | RL | 0.68 (0.05) | 0.69 (0.02) |
| 19 | f | 1.71 | 68.53 | RL | 0.69 (0.02) | 0.65 (0.02) |
| 20 | f | 1.7 | 72.98 | RL | 0.36 (0.02) | 0.31 (0.02) |
| 21 | f | 1.7 | 73.62 | RL | 0.3 (0.01) | 0.27 (0.01) |
| Group | - | 1.81 (0.1) | 80.21 (10.15) | - | 0.62 (0.16) | 0.58 (0.15) |

**Supplemental Document Table 2:** Participant number key to relate participant-specific findings from this paper to our prior published paper.

Liu, J. M., & Zaferiou, A. (2024). Whole-body linear momentum control in two-foot running jumps in male basketball players. Journal of Biomechanics, 175, 112300. <https://doi.org/10.1016/j.jbiomech.2024.112300>

| **This paper’s Participant #** | **Liu & Zaferiou 2024 Participant #** |
| --- | --- |
| 1 | 1 |
| 2 | 2 |
| 3 | 3 |
| 4 | 4 |
| 5 | Not Included |
| 6 | Not Included |
| 7 | 6 |
| 8 | Not Included |
| 9 | 7 |
| 10 | 8 |
| 11 | 9 |
| 12 | 10 |
| 13 | 11 |
| 14 | Not Included |
| 15 | 12 |
| 16 | 13 |
| 17 | 14 |
| 18 | Not Included |
| 19 | Not Included |
| 20 | Not Included |
| 21 | Not Included |

| **Var** | **Variable Name** | **TFRJs without a basketball** | | | **TFRJs with a basketball** | | |
| --- | --- | --- | --- | --- | --- | --- | --- |
|  |  | **Mean (S.D.)** | **r** | **p-value** | **Mean (S.D.)** | **r** | **p-value** |
| **Whole-body Kinematics Variables** | **Initial Downward COM Velocity (m/s)** | -0.84 (0.17) | 0.259 | 0.256 | -0.85 (0.12) | 0.225 | 0.326 |
| **COM Descent Subphase**  **Impulse Variables** | **Net Upward Impulse (Ns/kg)** | 0.67 (0.16) | -0.084 | 0.718 | 0.71 (0.12) | 0.031 | 0.895 |
|  | **Net Backward Impulse (Ns/kg)** | 0.92 (0.19) | 0.203 | 0.377 | 1.00 (0.20) | -0.015 | 0.949 |
|  | **First Leg Upward Impulse (Ns/kg)** | 1.95 (0.33) | -0.012 | 0.958 | 1.98 (0.41) | -0.222 | 0.332 |
|  | **First Leg Backward Impulse (Ns/kg)** | 0.89 (0.20) | 0.347 | 0.123 | 0.97 (0.21) | 0.171 | 0.46 |
| **COM Ascent Subphase**  **Impulse Variables** | **Net Upward Impulse (Ns/kg)** | 3.41 (0.49) | 0.987 | **<0.001** | 3.21 (0.47) | 0.931 | **<0.001** |
|  | **Net Backward Impulse (Ns/kg)** | 1.70 (0.37) | 0.803 | **<0.001** | 1.68 (0.38) | 0.832 | **<0.001** |
|  | **First Leg Upward Impulse (Ns/kg)** | 2.89 (0.41) | 0.672 | **<0.001** | 2.85 (0.46) | 0.574 | **0.007** |
|  | **Second Leg Upward Impulse (Ns/kg)** | 2.81 (0.41) | 0.69 | **<0.001** | 2.72 (0.42) | 0.651 | **0.002** |
|  | **First Leg Backward Impulse (Ns/kg)** | 0.52 (0.22) | 0.351 | 0.118 | 0.50 (0.21) | 0.34 | 0.132 |
|  | **Second Leg Backward Impulse (Ns/kg)** | 1.18 (0.32) | 0.663 | **0.001** | 1.19 (0.36) | 0.677 | **0.001** |
| **Temporal Variables** | **Total Ground Contact Duration (s)** | 0.37 (0.05) | -0.059 | 0.799 | 0.38 (0.05) | -0.198 | 0.39 |
|  | **COM Descent Subphase Duration (s)** | 0.14 (0.03) | -0.285 | 0.21 | 0.14 (0.04) | -0.24 | 0.293 |
|  | **COM Ascent Subphase Duration (s)** | 0.23 (0.03) | 0.192 | 0.403 | 0.24 (0.03) | 0.217 | 0.344 |
| **Average GRF through**  **Total Ground Contact Phase** | **First Leg Upward GRF (N/kg)** | 13.18 (1.50) | 0.546 | **0.01** | 12.99 (1.46) | 0.529 | **0.014** |
|  | **Second Leg Upward GRF (N/kg)** | 13.41 (1.61) | 0.83 | **<0.001** | 13.09 (2.00) | 0.827 | **<0.001** |
|  | **First Leg Backward GRF (N/kg)** | 3.89 (0.96) | 0.458 | **0.037** | 4.00 (0.80) | 0.459 | **0.036** |
|  | **Second Leg Backward GRF (N/kg)** | 5.67 (1.42) | 0.731 | **<0.001** | 5.76 (1.65) | 0.746 | **<0.001** |

**Supplemental Documents Table 3:** Group-level mean (± 1 standard-deviation), correlation coefficient (r), *p-value* of additional whole-body kinematics and kinetics variables against jump height in TFRJs with and without a basketball. *p-value­* bolded if significant (*α = 0.05*). Net upward impulse also accounted for the downward impulse due to body weight, such that it does not equal the sum of upward impulse generated by both legs.

**Supplemental Documents Table 4:** Group-level mean (± 1 standard-deviation), correlation coefficient (r), and *p-value* of the whole-body kinematics and impulse variables against jump height in TFRJs with and without a basketball for *male or female athletes separately*. There were 15 male participants and 6 female participants. *p-value­* bolded if significant (*α = 0.05*). Net upward impulse also accounted for the downward impulse due to body weight such that it does not equal to the sum of the upward impulse generated by both legs.

| **Variable Name** | **Sex** | **TFRJs without a basketball** | | | **TFRJs with a basketball** | | |
| --- | --- | --- | --- | --- | --- | --- | --- |
|  |  | **Mean (S.D.)** | **r** | **p-value** | **Mean (S.D.)** | **r** | **p-value** |
| **Jump Height (m)** | M | 0.62 (0.09) | - | - | 0.56 (0.08) | - | **-** |
|  | F | 0.35 (0.12) | - | - | 0.32 (0.11) | - | **-** |
| **Initial Forward COM Velocity (m/s)** | M | 3.99 (0.34) | 0.796 | **<0.001** | 4.05 (0.32) | 0.668 | **0.008** |
|  | F | 3.18 (0.49) | 0.798 | 0.057 | 3.29 (0.49) | 0.9 | **0.015** |
| **COM Ascent Distance (m)** | M | 0.46 (0.04) | 0.364 | 0.182 | 0.47 (0.04) | 0.425 | 0.115 |
|  | F | 0.38 (0.03) | 0.579 | 0.228 | 0.39 (0.03) | 0.321 | 0.535 |
| **Plant Angle (º)** | M | 57.25 (3.03) | -0.514 | **0.05** | 58.26 (2.78) | -0.481 | 0.07 |
|  | F | 62.54 (5.65) | -0.721 | 0.106 | 62.47 (4.44) | -0.849 | **0.033** |
| **Net Upward Impulse (Ns/kg)** | M | 4.33 (0.26) | 0.883 | **<0.001** | 4.16 (0.23) | 0.906 | **<0.001** |
|  | F | 3.43 (0.34) | 0.795 | 0.059 | 3.30 (0.36) | 0.926 | **0.008** |
| **First Leg Upward Impulse (Ns/kg)** | M | 5.04 (0.36) | -0.176 | 0.531 | 4.97 (0.34) | -0.173 | 0.538 |
|  | F | 4.35 (0.33) | 0.753 | 0.084 | 4.47 (0.61) | 0.165 | 0.755 |
| **Second Leg Upward Impulse (Ns/kg)** | M | 2.94 (0.37) | 0.543 | **0.039** | 2.84 (0.40) | 0.556 | **0.031** |
|  | F | 2.73 (0.43) | 0.209 | 0.691 | 2.63 (0.40) | 0.323 | 0.532 |
| **Net Backward Impulse (Ns/kg)** | M | 2.78 (0.36) | 0.471 | 0.078 | 2.81 (0.33) | 0.617 | **0.014** |
|  | F | 2.22 (0.43) | 0.555 | 0.253 | 2.37 (0.46) | 0.737 | 0.094 |
| **First Leg Backward Impulse (Ns/kg)** | M | 1.51 (0.23) | 0.215 | 0.441 | 1.54 (0.17) | 0.121 | 0.668 |
|  | F | 1.17 (0.25) | 0.09 | 0.865 | 1.30 (0.22) | 0.287 | 0.581 |
| **Second Leg Backward Impulse (Ns/kg)** | M | 1.27 (0.28) | 0.528 | **0.043** | 1.27 (0.32) | 0.583 | **0.023** |
|  | F | 1.05 (0.32) | 0.675 | 0.141 | 1.07 (0.38) | 0.143 | 0.803 |

**Supplemental Document Table 5:** Participant post-data collection interview questions and responses. The interviews are only conducted from participant 11 onward.

| **Participant Number** | **Basketball Experience** | **How did you learn to jump?** | **Have you been told or coached to jump?** | **Have you watched videos or received suggestions about jump technique** | **Do you practice jumping with or without ball?** | **Have you been specifically asked to practice jumping with or without ball?** |
| --- | --- | --- | --- | --- | --- | --- |
| **11** | College varsity. | Father taught “pendulum” arm swing; practice from playing. | Yes, coached on jumping technique (transitioning from 1-foot to 2-foot). | Yes—especially to learn 2-foot jump form (arm swing, staying low). | Mostly without ball (once/twice a week). | No. |
| **12** | No formal team. | Self-taught via “dunk training.” | Yes—e.g., “lengthen penultimate,” “don’t drop hips.” | Yes (implied, though not deeply elaborated beyond penultimate/hips advice). | Yes—2-foot jumps once every ~2 weeks. | Not specifically told, but does both. |
| **13** | High school varsity. | Inspired by online dunk videos; had a jump coach from 2020–2021. | Yes—received jump coaching, learned about periodization, some strength/plyo approaches. | Yes—online tutorials, learned deadlifts, plyometrics, approach jumps. | ~6–8 hours/week w/o ball, ~1 hour/week with ball. | No. |
| **14** | Collegiate level. | No formal coaching—just did it naturally. | Yes—general instructions (bend knees, foot plant). | Yes—on bending knees, swinging arms. | No-ball box jumps in weight room; with-ball jumps in team practices. | No—does what is asked in the weight room/practice. |
| **15** | High school varsity (in China). | Learned high jump first (elementary school), adapted it to basketball. | Only for high jump, not specifically for basketball. | Yes—YouTube and TikTok. | ~70% with ball, ~30% without. | Sometimes told, but not very frequent. |

**Supplemental Document Table 5 continued**

| **16** | D3 college basketball. | Obsessed from a young age, found a coach; trained a lot. | Yes—various coaches giving technique tips. | Yes—lots of resources on approach speed, arm swing, body angles. | A lot with ball (games); used to do ~10–20 no-ball jumps/day. | Yes—some coaches explicitly separated each. |
| --- | --- | --- | --- | --- | --- | --- |
| **17** | Intramural basketball | Through playing volleyball | Yes—playing volleyball | Yes—long penultimate step, arm swing, block foot action, center of mass positioning | Yes—once a week | Yes. |
| **18** | High school varsity | Just do basic jumping | No. | Yes—some videos on tik tok and youtube, on arm swing | Yes—Usually jump with a ball when playing basketball | No. |
| **19** | High school varsity with D1 offer | Natural jumper and track and field | No. | **Yes**—arm swing, ankle mobility, jumping vertically and angles | Yes—once a week | **No.** |
| **20** | D3 college basketball | Mostly Box jumps for increasing vertical | No. | No. | No—Only jumps without a ball (weighted jump), no jumps with a basketball | No |
| **21** | D3 college basketball | Natural | Yes—Only foot work on layups | No. | No—box jumps without a ball | No. |
